# Granular Ophthalmia

**Published:** 1891-02-28

**Authors:** 


### OPHTHALMATOLOGY.

### GRANULAR OPHTHALMIA.

This disease used to be regarded as certainly one of the most tedious affections of the eye, proving in many cases almost incurable. Until a few years ago almost the only recognised treatment consisted in the application of caustics and astringents to the granulations on the everted lids. The lids certainly did get well if the treatment was persisted in, but how tedious and painful this process of cure was none but the patient can realise. The two most important items which have led to the improved treatment of trachoma are, first a knowledge of the cause—that it is contagious, depending on a trachomacoccus, and in nearly all cases occurring in eyes that have been attacked previously, by acute purulent or muco-purulent ophthalmia. Secondly, the discovery of cocaine has enabled the surgeon to carry out the treatment more thoroughly. The disease can now be treated without much pain, and in most cases effectually, by the mitigated nitrate of silver stick, used three or four times a week, supplemented by the use of instillations of sulphate of copper. But there are a certain number of cases in which such simple treatment is ineffectual. The treatment of such cases has of late years become more energetic. For a time pure carbolic acid, carefully applied with a brush, had its advocates, and certainly it acts very well in many cases, and is generally not so painful as nitrate of silver. Its application is often followed by a good deal of swelling of the lids, but this soon subsides. More recently powdered boric acid has been tried; a considerable quantity is vigorously rubbed on the granulations daily. In a good many cases in which we have tried it the results were decidedly inferior to those obtained with the use of nitrate of silver. Electrolysis and the galvanocautery have also been recommended. Both are good in so far as they destroy the trachoma tissue, but electrolysis needs the special apparatus, and its success depends in part at least on its also having a cauterising effect, whilst the galvanocautery is just as efficacious and easier to apply. It is particularly suitable when the granulations are prominent, but some avoid using it on the grounds that the resulting cicatrix is likely to contract and cause entropion. The probability of this is not nearly so great as that entropion will result if the trachoma is not soon cured. Another remedy that is highly spoken of by Arnaut is the application of a solution of perchloride of mercury (1 in 120) twice a-week, with a weaker solution (1 in 500) to be dropped into the eye three times a-day. We have had no experience of this treatment, but should imagine that the drops would be very painful, as patients using a lotion only one-eighth of the strength often complain of the smarting being considerable. Lastly, what may be called a radical cure has been introduced. Its success depends on its thoroughness, the object being to entirely destroy all the granulations and trachomacocci. The operation is performed under an anæsthetic. The outer canthus is freely divided with scissors, so that the upper conjunctival *cul-de-sac* may be entirely exposed. Each mass of granulation is cut across with a knife, and the granulation tissue scraped away with a curette or squeezed out with a pair of smooth-ended curved forceps. The next process is to irrigate the surface with a lotion of perchloride of mercury (1 in 500), and at the same time to brush it very thoroughly with a stiff brush, so as to remove every particle of trachoma tissue. Compresses wet with solution of perchloride of mercury are kept applied, and on the third day the lids are again everted and mopped with a solution of 1 in 500. It is too soon to speak with certainty of the permanence of the results obtained by this method. Obviously it is only adapted to the most obstinate cases, and in them a process of cicatrization may have been set up before the *curettage* was resorted to; and so it is not unlikely that in some instances a contracting scar with entropion will follow. But the results are so far promising that it ought to be adopted after nitrate of silver, carbolic acid, and the galvanocautery have successively had fair trial.

